# Vertically Ordered Mesoporous Silica-Nanochannel Film-Equipped Three-Dimensional Macroporous Graphene as Sensitive Electrochemiluminescence Platform

**DOI:** 10.3389/fchem.2021.770512

**Published:** 2021-11-22

**Authors:** Jiawei Gong, Hongliang Tang, Xuan Luo, Huaxu Zhou, Xueting Lin, Kailong Wang, Fei Yan, Fengna Xi, Jiyang Liu

**Affiliations:** ^1^ Department of Chemistry, Key Laboratory of Surface and Interface Science of Polymer Materials of Zhejiang Province, Zhejiang Sci-Tech University, Hangzhou, China; ^2^ Affiliated Fangchenggang Hospital, Guangxi University of Chinese Medicine, Fangchenggang, China; ^3^ The First Clinical Faculty of Guangxi University of Chinese Medicine, Nanning, China; ^4^ The First Affiliated Hospital of Guangxi University of Chinese Medicine, Nanning, China

**Keywords:** three-dimensional graphene, electrochemiluminescence sensor, vertically ordered mesoporous silica-nanochannel film, 4-chlorophenol, chlorpheniramine

## Abstract

Three-dimensional (3D) electrochemiluminescence (ECL) platform with high sensitivity and good anti-fouling is highly desirable for direct and sensitive analysis of complex samples. Herein, a novel ECL-sensing platform is demonstrated based on the equipment of vertically ordered mesoporous silica-nanochannel films (VMSF) on monolithic and macroporous 3D graphene (3DG). Through electrografting of 3-aminopropyltriethoxysilane (APTES) onto 3DG as molecular glue, VMSF grown by electrochemically assisted self-assembly (EASA) method fully covers 3DG surface and displays high stability. The developed VMSF/APTES/3DG sensor exhibits highly sensitized ECL response of tris(2,2′-bipyridyl) ruthenium (Ru (bpy)_3_^2+^) taking advantages of the unique characteristics of 3DG (high active area and conductivity) and VMSF nanochannels (strong electrostatic enrichment). The VMSF/APTES/3DG sensor is applied to sensitively detect an important environmental pollutant (4-chlorophenol, with limit of detection or LOD of 30.3 nM) in term of its quenching effect (ECL signal-off mode) toward ECL of Ru (bpy)_3_^2+^/tri-n-propylamine (TPrA). The VMSF/APTES/3DG sensor can also sensitively detect the most effective antihistamines chlorpheniramine (with LOD of 430 nM) using ECL signal-on mode because it acts as co-reactant to promote the ECL of Ru (bpy)_3_^2+^. Combined with the excellent antifouling ability of VMSF, the sensor can also realize the analysis of actual environmental (lake water) and pharmaceutical (pharmacy tablet) samples. The proposed 3D ECL sensor may open new avenues to develop highly sensitive ECL-sensing platform.

## Introduction

Solid-state nanofilms have recently been widely used in the fields of molecular sieves, energy conversion, nanofluidics, and biosensing owing to their adjustable nanopores, intelligent control of molecular transport, and high device integration capabilities (Huang et al., 2016; Dong et al., 2020; Garbayo et al., 2021). The vertically ordered mesoporous silica-nanochannel thin film (VMSF) has attracted extensive attentions because of its unique characteristics (Nasir et al., 2016; Chen et al., 2019; Ullah et al., 2020; Wang et al., 2020). In general, VMSF has well-ordered (regular hexagonal packing) and vertically aligned nanochannel arrays with uniform diameter (usually 2–3 nm), high density (more than 10^12^ pores/cm^2^), and adjustable thickness (usually 50–200 nm) (Yan et al., 2016; Wang et al., 2017; Ding et al., 2020a; Zhou et al., 2020a; Ding et al., 2020b; Ullah et al., 2021). In addition to the high specific surface area, chemical and mechanical stability of mesoporous materials, VMSF also exhibits high selectivity for molecular size and charge. On the one hand, derived from its silanol groups (p*K*_a_ ∼ 2), VMSF usually has an anionic nature under normal pH conditions. This charge characteristic accelerates the transfer of positively charged molecules to the electrode surface, thereby improving the detection sensitivity. In addition, VMSF shows good anti-fouling ability because the ultrasmall nanochannels can effectively inhibit the interference of co-existing large substances (e.g., proteins), leading to good signal stability in analysis of complex samples (e.g., whole blood, biological fluids, environmental, or food samples) (Yan and Su, 2016; Zhou et al., 2018; Duan et al., 2021; Gamero-Quijano et al., 2021). Thus, electrode equipped with VMSF shows great potential in direct and sensitive detection of complex samples owing to significant anti-fouling and signal amplification abilities of VMSF.

Electroluminescence (ECL) is the luminescence caused by the electron transfer of reactants at an electrode surface under the excitation of electrochemistry (Cheng et al., 2020; Gu et al., 2020; Ma et al., 2020). Compared with luminescence technologies such as fluorescence and chemiluminescence, ECL does not need excitation light and, therefore, has the advantages of low background and high signal-to-noise ratio. At the same time, the luminescence reaction of ECL is strictly controlled by electrochemical excitation and occurs in the diffusion layer adjacent to the electrode surface, leading to controllable reaction and high sensitivity. Up to now, ECL has become a powerful tool for immunoassay, DNA or cell analysis, environmental analysis, etc. (Liu et al., 2015; Li et al., 2017; Zhang et al., 2019). Among a large number of organic or inorganic ECL luminophores, tris(2,2′-bipyridine) ruthenium (Ru (bpy)_3_^2+^) is the most extensively used in scientific research and commercial applications because of its good chemical stability, high electrochemiluminescence quantum yield, and water solubility (Ding et al., 2020b; Duan et al., 2021). Owing to the strong electrostatic attraction and accelerated mass transfer of the negatively charged VMSF nanochannel arrays to positively charged Ru (bpy)_3_^2+^, VMSF-modified electrodes promise great potential in highly sensitive ECL detection.

Until now, most VMSF-based sensors are mainly using indium tin oxide (ITO) as the supporting electrodes, essentially because the hydroxyl groups on ITO surface ensure good mechanical stability of VMSF through the formation of Si–O covalent bond between VMSF and the electrode surface. For carbon or gold electrodes that have poor adhesion with VMSF, organosilane or specific nanomaterials are needed as molecular glue or adhesive layer to pre-modify the electrode surface to improve the stability of VMSF. For instance, the Walcarius group proposed to improve the adhesion of VMSF on glassy carbon electrode by electrografting 3-aminopropyltriethoxysilane (APTES) on the electrode (Nasir et al., 2016). They also assembled 3-mercaptopropyl (trimethoxysilane) on gold electrode as a molecular glue to grow stable VMSF (Ullah et al., 2020). Our group demonstrated the use of reduced graphene oxide (rGO) sheets as conductive adhesive and electroactive layer for growing stable VMSF on GCE (Xi et al., 2019). In addition to electrode materials, electrode structure is also critical to the performance of electroanalysis. Compared with the traditional 2D planar electrode, three-dimensional (3D) porous electrode has high surface area and excellent diffusion/mass transfer, which can effectively improve the detection sensitivity (Huang et al., 2020; Beluomini et al., 2021; He et al., 2021). Up to now, however, a stable equipment of VMSF on 3D porous electrode for the development of highly sensitive ECL sensor has not been reported.

**FIGURE 1:**
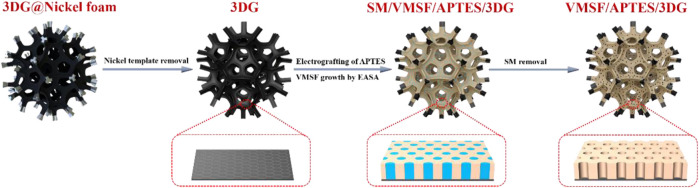
Schematic illustration for the preparation of vertically ordered mesoporous silica-nanochannel films (VMSFs)/(3-aminopropyl) trimethoxysilane (APTES)/three-dimensional grapheme (3DG).

In this work, we present a novel 3D ECL sensing platform based on the equipment of VMSF on porous 3D graphene foam electrode (3DG). The 3DG is grown by chemical vapor deposition (CVD) using Ni foam as template and, therefore, has monolithic and macroporous structure. Through the electrografting of APTES onto the surface of 3DG as molecular glue, stable VMSF is conveniently grown on the 3D electrode using electrochemically assisted self-assembly (EASA) method (Figure 1). The feasibility and universality of the ECL sensor (VMSF/APTES/3DG) is validated by the detection of an important environmental pollutant (4-chlorophenol) and a common antihistamine drug (chlorpheniramine) as the proof-of-concept demonstrations. A signal-off ECL mode is developed to detect 4-chlorophenol based on its quenching effect on the ECL system of Ru (bpy)_3_^2+^/TPrA. On the contrary, the chlorpheniramine can act as a co-reactant to enhance the ECL of Ru (bpy)_3_^2+^, so it can be detected in a signal-on ECL mode. Owing to the good conductivity and high active surface of 3DG, and the significant enrichment effect of VMSF nanochannels on the ECL probe of Ru (bpy)_3_^2+^, VMSF/APTES/3DG sensor exhibits high sensitivity and low detection limits. Thanks to the excellent anti-fouling ability of VMSF, VMSF/APTES/3DG sensors that worked well in the analysis of real environmental or pharmaceutical samples.

## Materials and Methods

### Chemicals and Materials

Tetraethoxysilane (TEOS), tris(2,2-bipyridyl) dichlororuthenium (II) hexahydrate (Ru (bpy)_3_Cl_2_ 6H_2_O), cetyltrimethylammonium bromide (CTAB), and hexaammineruthenium (III) chloride (Ru(NH_3_)_6_Cl_3_) were purchased from Sigma-Aldrich (USA). Tripropylamine (TPrA), 4-chlorophenol (4-CP), potassium ferricyanide (_K3_ [Fe(CN)_6_]), (3-aminopropyl) trimethoxysilane (APTES), tetrabutylammonium tetrafluoroborate (Bu_4_N^+^BF_4_^−^), and acetonitrile were obtained from Aladdin (China). Lake water sample was obtained from the campus lake of Zhejiang Sci-Tech University (Hangzhou, China). Chlorphenamine maleate tablets were purchased from Tianjin Lisheng Pharmaceutical Co., Ltd (China). All other chemicals were of analytical grade. Ultrapure water (18.2 MΩ cm) was used throughout the work.

### Preparation of Three-Dimensional Graphene Electrode

As previously reported (Xi et al., 2013), 3DG was synthesized by CVD using nickel foam as the template and ethanol as the precursor. Subsequently, the nickel substrate was etched away with 3 M HCl solution overnight at 80°C to obtain the free-standing 3DG. To prepare electrode, 3DG (3 × 8 mm, 1-mm thick) was fixed onto a glass slide. Electrode wire was made by connection copper wire and 3DG by silver glue. Then, copper wire and silver glue were insulated with silicone rubber.

### Fabrication of Vertically Ordered Mesoporous Silica-Nanochannel Films /(3-Aminopropyl) Trimethoxysilane /3D Grapheme Sensor

The fabrication of VMSF/APTES/3DG sensor included three steps. First, APTES is electrografted onto the surface of 3DG as a molecular glue to improve the mechanical stability of VMSF according to a published procedure (Nasir et al., 2016). Briefly, 3DG electrode was placed in an acetonitrile solution containing APTES (1.0 mM) and tetrabutylammonium tetrafluoroborate (Bu_4_N^+^BF_4_^−^, 0.1 M). The electrografting of APTES was achieved through electrochemical oxidation of aliphatic amines by 5-circle scan using cyclic voltammetry (CV, scan rate was 100 mV/s, potential range was 0.7–2.0 V). The obtained APTES/3DG was then thoroughly rinsed with acetone. Second, EASA method was used to prepare surfactant-containing VMSF/APTES/3DG using surfactant micelles (SM) as a template (Nasir et al., 2016). Typically, NaNO_3_ solution (0.1 M, pH = 2.6) and ethanol was mixed at a ratio of 1:1 (v/v) before CTAB (32 mM) and TEOS (100 mM) were added. The resulting precursor solution was pre-hydrolyzed under stirring at room temperature for 2.5 h. Then, the APTES/3DG was immersed in the above electrodeposition medium and a current density of −0.74 mA/cm^2^ was applied for 10 s for the growth of VMSF. The resulting electrode was quickly taken out followed with thorough rinse with water and drying with N_2_ stream, respectively. After being aged at 80°C overnight to cross-link the silica network, VSMF modified electrode with SM in nanochannels was obtained and designated as SMs@VMSF/APTES/3DG. Finally, SM was removed by incubating the electrode in a mixture of HCl (0.1 M) and ethanol solution (1/1, v/v) under stirring for 5 min, leading to the final VMSF/APTES/3DG electrode with open nanochannels. For comparison, VMSF modified GCE electrode (VMSF/APTES/GCE) was prepared using the same procedure with GCE as the supporting electrode.

### Measurements and Instrumentations

Transmission electron microscopy (TEM) images were obtained on copper grid at 100 kV using a HT7700 microscope (Hitachi, Japan). The morphology of the bare or modified 3D electrodes and the chemical composition at the sample surface were characterized by field emission scanning electron microscopy (SEM, HT7700, Hitachi, Japan) equipped with an energy dispersive spectrometer (EDS, Bruker Nano Xflash610-H, Germany). Electrochemical measurements were carried out on an Autolab PGSTAT302N potentiostat (Metrohm, Switzerland). A conventional three-electrode system was adopted. Briefly, bare or modified 3DG or GCE was the working electrode. Ag/AgCl electrode (saturated with KCl) was used as the reference electrode and Pt sheet (1 × 1 cm) was the counter electrode. ECL intensity measurements were performed on the MPI-E ECL analytical system (Remex Analysis Instrument, Xi’an, China). The photomultiplier tube (PMT) biased at 600 V.

### Electroluminescence Determination of 4-Chlorophenol

To detect 4-chlorophenol, phosphate buffered saline (PBS, 0.01 M, pH 7.4) containing Ru (bpy)_3_^2+^ (10 μM) and TPrA (3 mM) was used as the supporting solution. After different concentrations of 4-chlorophenol was added, the VMSF/APTES/3DG electrode was scanned using CV method (potential range: 0–1.5 V, scan rate: 100 mV/s). The corresponding ECL signal generated during CV scan was recorded. For real sample analysis, the standard addition method was used to evaluate the reliability of the developed ECL sensor. The lake water was filtered using Nylon microfiltration membrane (0.22 μm) and then diluted by a factor of 20 with PBS (0.01 M, pH 7.4). ECL detection was carried out after adding a certain amount of 4-chlorophenol.

### Electroluminescence Determination of Chlorpheniramine

For the detection of chlorpheniramine, PBS (0.01 M, pH 7.4) containing10 μM Ru (bpy)_3_^2+^ was applied as the medium. After different concentrations of chlorpheniramine were added, ECL signal of the solution was measured under CV scanning (potential range: 0–1.5 V, scan rate: 100 mV/s). To analyze chlorpheniramine in pharmacy tablets, the tablets were grinded and dissolved in PBS (0.01 M, pH 7.4). The resulting solution was centrifuged at 7,000 rpm for 5 min to remove insoluble starch and the supernatant was analyzed using VMSF/APTES/3DG sensor.

## Results and Discussion

### Convenient Equipment of Vertically Ordered Mesoporous Silica-Nanochemical Films on Three-Dimensional Grapheme Electrode

**FIGURE 2:**
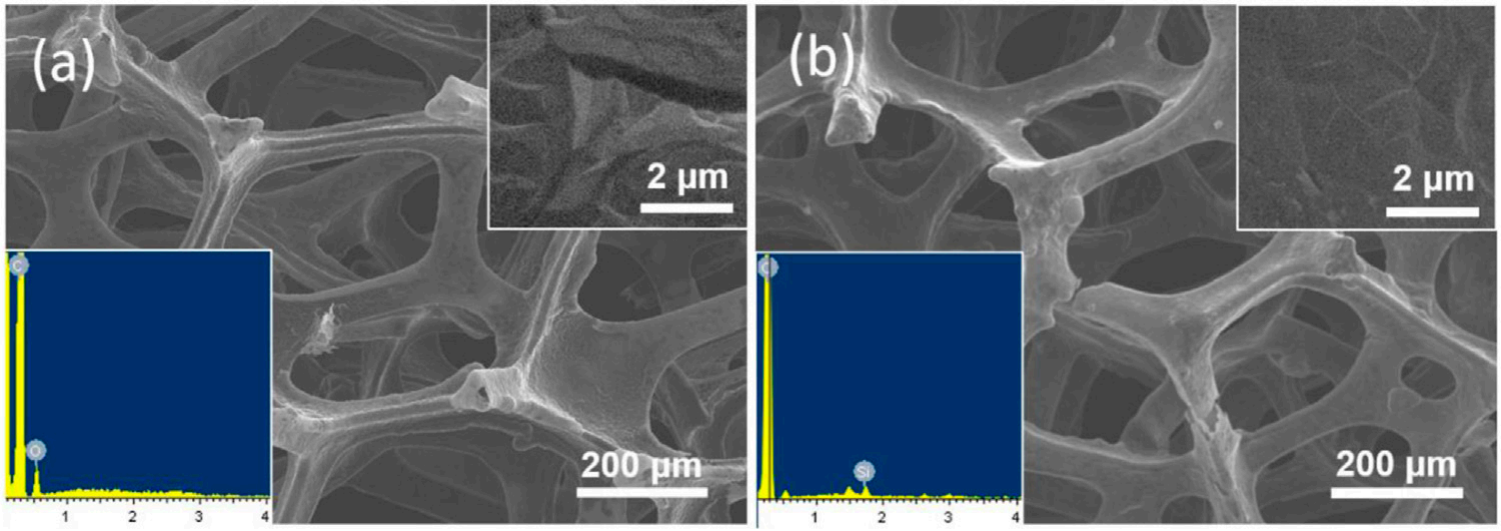
SEM images of **(A)** bare 3DG and **(B)** VMSF/APTES/3DG at different magnifications. Inset in (a) and (b) is the corresponding energy dispersive spectrometer (EDS) spectrum.

In present work, the equipment of VMSF on 3D electrode is first demonstrated. The 3D monolithic graphene foam (3DG) served as the supporting electrode. Figure 1 illustrates the convenient growth of VMSF after electrografting APTES on 3DG. Figure 2A shows SEM images of 3DG grown by CVD under different magnifications. As shown, bare 3DG presents a monolithic structure with well-defined macropores. The surface of the graphene skeleton is smooth, and the wrinkled structure of graphene can be obviously observed. The energy dispersive spectrometer (EDS) characterization shows high content of C atoms (inset in Figure 2A, the O signals result from oxygen in macropores of 3DG). Compared with other porous 3D graphene assembled from rGO sheets, 3DG has interconnected skeleton comprised of defect-free graphene, allowing rapid charge and mass transfer. In addition to the high conductivity of CVD-grown graphene, 3DG electrode also exhibits large active area and unhindered substance diffusion in comparison with 2D planar electrodes (Li et al., 2019). However, the high hydrophobicity of graphene foam brings difficulties to its functionalization and application.

After the direct growth of VMSF on 3DG, the resulted VMSF/3DG electrode presents uneven white areas visible to the naked eye, indicating that the electrode surface was not completely covered by VMSF. This was attributed to the poor wettability of 3DG in film forming solutions caused by its high hydrophobicity during the film growth process. In addition, the white areas disappeared after rinsing with water, suggesting the poor adhesion of VMSF to the unmodified 3DG substrate. Thus, electrografting APTES on the surface of 3DG as hydrophilic molecular glue is used to overcome these two problems. On the one hand, the electrografting medium contains organic solvent acetonitrile, which can ensure the spread of the solution on the surface of 3DG. On the other hand, APTES could be electrografted to carbon surface by forming C-N covalent bond through oxidizing its amine groups. At the same time, three silane groups of APTES are able to anchor VMSF (Nasir et al., 2016; Nasir et al., 2018; Jiokeng et al., 2019; Nasir et al., 2019; Zhou et al., 2020b; Nasir et al., 2020). Therefore, APTES can function as an effective molecular glue to improve the adhesion of VMSF to the surface of 3DG.

VMSF can be quickly and conveniently deposited onto APTES/3DG electrode by electrochemically assisted self-assembly (EASA) method. Through applying a cathode potential to the APTES/3DG electrode, the reduction of protons on the electrode causes a local increase in the pH value adjacent to the electrode surface. The generated hydroxyl ions (OH^−^) at the electrode/solution interface could serve as catalysts to induce the condensation of TEOS around SM by self-assembly, leading to concomitant growth of VMSF on the electrode.

SEM images of VMSF/APTES/3DG electrode at different magnification are shown in Figure 2B. As revealed, the morphology of 3DG hardly changed after modification with APTES owing to the characteristic of molecular glue. After VMSF was subsequently equipped, there was still no significant change in morphology of 3DG because of the nanofilm structure. However, EDS characterization shows that Si groups appear on the surface of VMSF/APTES/3DG electrode, indicating the effective combination of VMSF (inset in Figure 2B).

### Characterization of Vertically Ordered Mesoporous Silica-Nanochannel Film and Vertically Ordered Mesoporous Silica-Nanochannel Film/(3-Aminopropyl) Trimethoxysilane/Three-Dimensional Graphene Electrode

**FIGURE 3:**
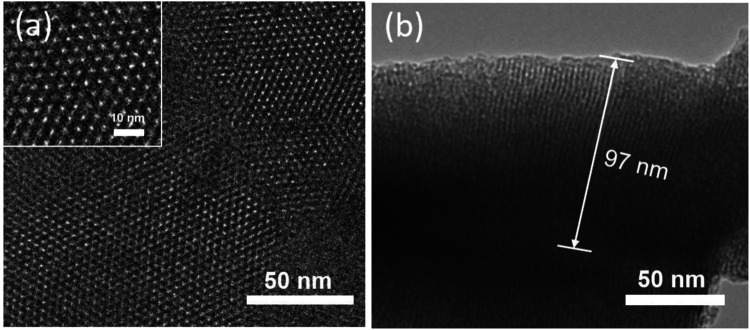
**(A)** Top view of transmission electron microscopy (TEM) images of VMSF. **(B)** Cross-section view of TEM image of VMSF. Inset in (a) shows the magnified view.

The structure and morphology of the prepared VMSF were characterized by TEM. Figure 3 shows TEM images of VMSF detached from APTES/3DG electrode. The top view (Figure 3A) reveals the hexagonal compact arrangement of mesopores with an aperture of approximately 2.2 nm (inset in Figure 3A). In addition, the film is uniform over a wide area. The cross section (Figure 3B) demonstrates the mesopore nanochannels possess long-range ordered structure with an average length of around 97 nm.

**FIGURE 4:**
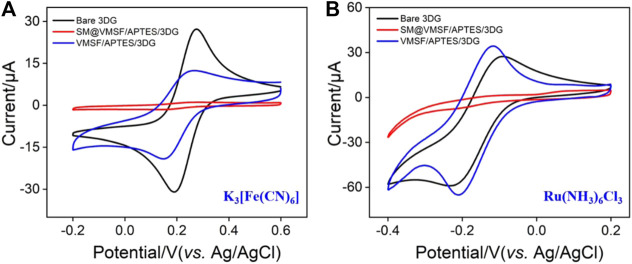
CV curves obtained at bare 3DG, SM@VMSF/APTES/3DG, and VMSF/APTES/3DG in 0.05 M KHP solution containing 0.5 mM K_3_ [Fe(CN)_6_] **(A)** and 0.5 mM hexaammineruthenium III chloride (Ru(NH_3_)_6_Cl_3)_ **(B)**. The scan rate was 50 mV s^−1^.

The integrity, permeability, and charge permselectivity of VMSF/APTES/3DG were investigated by cyclic voltammetry (CV). For comparison, bare 3DG and SM@3DG/APTES/3DG were also examined. Figure 4 shows the CV curves of standard redox probes including anionic Fe(CN)_6_^3−^ and Ru(NH_3_)_6_^3+^ at the three types of electrodes. Bare 3DG exhibits well-defined redox peaks owing to the high conductivity of graphene. Due to the presence of templated SM inside the nanochannels of VMSF, the SM@VMSF/APTES/3DG electrode shows almost no electrochemical responses toward the two redox molecules, indicating that the SM inside the nanochannels prevents the charged probes from contacting the substrate electrode. This phenomenon also proves the good adhesion and full coverage of VMSF on the APTES/3DG electrode without cracks. In contrast, SM@VMSF/3DG electrode that was prepared without APTES displayed an obvious redox peak, proving low adhesion of VMSF on 3DG (Supplementary Figure S1 in Supplementary material). After removal of the SM template from the nanochannels, the obtained VMSF/APTES/3DG electrode exhibits definite electrochemical signals, indicating the efficient diffusion of the redox probes through the nanochannels and the electron transfer with the supporting electrode.

Owing to the negatively charged nanochannels (the p*K*_a_ of silica is ∼2), VMSF exhibits strong effect on the mass transfer of the charged molecules through electrostatic interactions. On the one hand, the signal of Fe(CN)_6_^3−^ at VMSF/APTES/3DG is lower than that at bare 3DG due to the electrostatic repelling of VMSF nanochannels. In case of Ru(NH_3_)_6_^3+^, on the other hand, the response on VMSF/APTES/3DG is even higher than that on bare 3DG, suggesting that negatively charged VMSF is quite beneficial to enrich the positive probes.

### Enhanced Electrochemiluminescence of tris(2,2’-Bipyridine Ruthenium by Vertically Ordered Mesoporous Silica-Nanochannel Film

**FIGURE 5:**
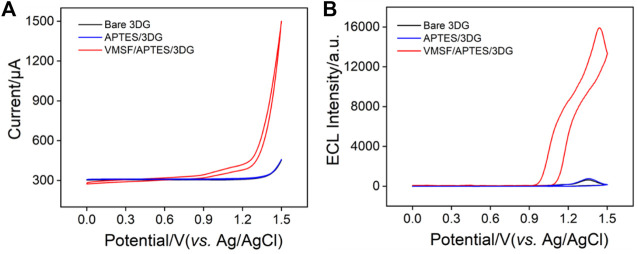
**(A)** CV or **(B)** electroluminescence (ECL) curves obtained at bare 3DG, APTES/3DG, and VMSF/APTES/3DG in phosphate buffered saline (PBS) (0.01 M, pH 7.4) containing tris(2,2′-bipyridine) ruthenium (Ru (bpy)_3_^2+)^ (10 μM) and tripropylamine (TPrA) (3 mM). The scan rate was 100 mV s^−1^.

Owing to the negatively charged nanochannels surface, VMSF can significantly enrich positively charged species and facilitate their mass transport *via* strong electrostatic interactions (Zhou et al., 2015; Liu et al., 2016; Guo et al., 2016; Xiao et al., 2017). Thus, the enhanced electrochemiluminescence (ECL) of Ru (bpy)_3_^2+^ on VMSF/APTES/3DG during CV scan was investigated using TPrA as the co-reactant. For comparison, the ECL signals on bare 3DG or APTES/3DG were also measured. As shown in Figure 5, 3DG and APTES/3DG exhibit very close CV and ECL signals, indicating that APTES modification did not significantly change the electrode performance of 3DG. In addition to modifying 3DG with APTES to improve its hydrophilicity and promote VMSF adhesion, we also tried to treat 3DG with oxygen plasma to achieve the same purpose. The obtained electrode is designated as pl-3DG. As known, oxygen plasma treatment of carbon materials can introduce oxygen-containing groups (e.g., −OH groups) and improve their hydrophilicity. The generated oxygen-containing groups are also conducive to the stable adhesion with VMSF. However, we found that the ECL signal of pl-3DG was 50% lower than that of bare 3DG due to the quench effect of the surface oxygen-containing groups on the excited luminophore Ru (bpy)_3_^2+*^ (Zu and Bard, 2000; Qin et al., 2020). Thus, modification of 3DG with APTES is quite suitable to integrate VMSF for further construction of the 3D ECL sensor.

In the case of VMSF/APTES/3DG, a remarkable anodic current wave is revealed at potentials more positive than 0.9 V (Figure 5A). At the same time, the corresponding ECL signal is significantly enhanced (Figure 5B). The ECL intensity is about 25 times higher than that of bare 3DG. The remarkably sensitized ECL signal is attributed to the strong enrichment effect by the negatively charged nanochannel of VMSF on the cationic Ru (bpy)_3_^2+^. To further confirm the sensitization effect of VMSF matrix on the ECL signal of Ru (bpy)_3_^2+^, we used the same method to modify the traditional 2D planar electrode, glassy carbon electrode (GCE). Compared with bare GCE, the obtained VMSF/APTES/GCE shows a remarkably improved ECL signal, confirming the universality of VMSF-sensitized ECL signal (Supplementary Figure S2 in Supplementary material). In addition, VMSF/APTES/3DG shows a significantly higher ECL signal than VMSF/APTES/GCE, indicating that 3DG electrode with larger active electrode area can effectively enhance ECL signal. Thus, a high ECL signal can be obtained at a low Ru (bpy)_3_^2+^ concentration (10 μM). This can significantly reduce the cost of ECL analysis because Ru (bpy)_3_^2+^ is expensive. In addition, VMSF/APTES/3DG has great potential in the fabrication of ECL platform with high sensitivity.

### The Stability, Reproducibility and Anti-Fouling Abilities of Vertically Ordered Mesoporous Silica-Nanochannel Film/(3-Aminopropyl) Trimethoxysilane/Three-Dimensional Graphene Electrode

To evaluate the ECL stability, time-dependent ECL signals of VMSF/APTES/3DG electrode in Ru (bpy)_3_^2+^/TPrA solution during successive CV scans were investigated. As shown in Supplementary Figure S3 (Supplementary material), a relative standard deviation (RSD) of 1.6% was observed for 11th measurements, indicating high ECL stability. The reproducibility of the electrode was studied by preparing five electrodes in parallel under the same conditions. The RSD of the ECL signal was 2.5%, confirming high reproducibility. After the three VMSF/APTES/3DG electrodes were stored in the refrigerator (4°C) for 2 weeks, the RSD of 2.7% was observed. Thus, VMSF/APTES/3DG electrode has good operational stability and reproducibility.

The anti-fouling ability of the electrode is very important because electrode fouling leads to low accuracy and sensitivity. To evaluate the anti-fouling ability of VMSF/APTES/3DG, starch and humic acid, that are abundant in medical tablets or environmental water sample, are chosen as the investigated matrices. The ECL intensity of Ru (bpy)_3_^2+^/TPrA at VMSF/APTES/3DG in the presence of starch or humic acid was compared with that at bare 3DG electrode (Supplementary Figure S4 in Supplementary material). As shown, the ECL intensity on bare 3DG electrode is significantly reduced in the presence of starch or humic acid. On the contrary, no remarkable change in ECL intensity is observed for VMSF/APTES/3DG, indicating high anti-fouling ability.

### Electrochemiluminescence Determination of 4-Chlorophenol Using Signal-off Mode

**FIGURE 6:**
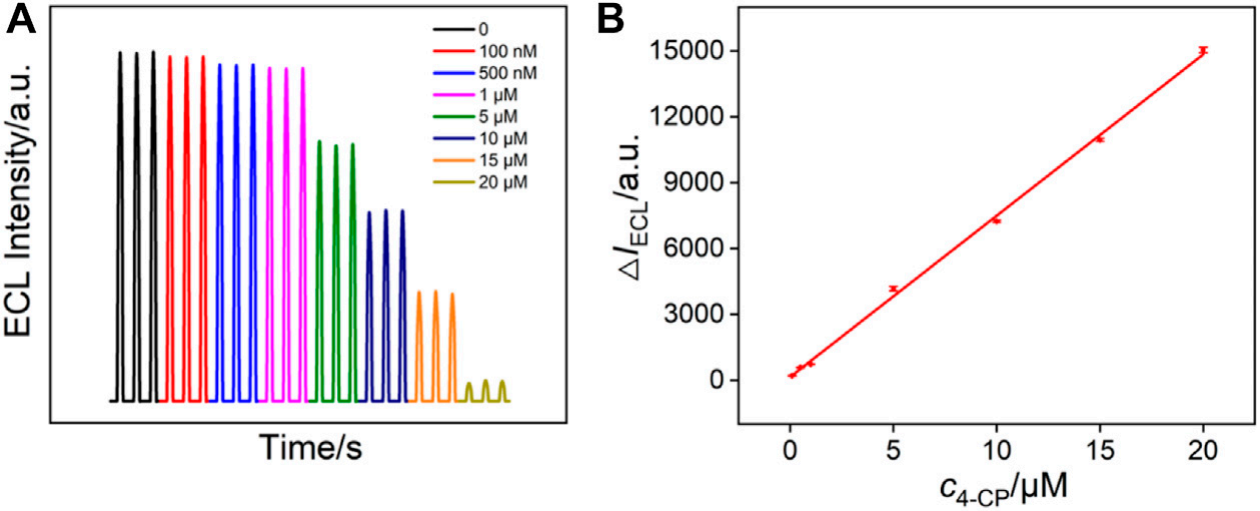
**(A)** ECL intensity of Ru (bpy)_3_^2+^/TPrA system in the presence of different concentrations of 4-chlorophenol. **(B)** The linear dependence between the reduced ECL intensity (ΔI_ECL_) and the concentration of 4-chlorophenol.

Through monitoring the quenching effects of analytes on the ECL system, ECL signal-off detection mode can be realized. As a proof-of-concept demonstration, ECL generated by the ECL system of Ru (bpy)_3_^2+^/TPrA on VMSF/APTES/3DG was employed to detect 4-chlorophenol (4-CP) in term of its quenching effect (Li et al., 2015; Qi et al., 2017). Generally, 4-chlorophenol is released into the environment as a by-product of paper bleaching, drinking water disinfection, petroleum industry, and other industries, causing serious pollution in aquatic and terrestrial ecosystems. As 4-chlorophenol itself is relatively stable to photodegradation, so its environmental residence time is relatively long. Therefore, both the US Environmental Protection Agency (EPA) and the European Union (EU) have marked 4-chlorophenol as a priority pollutant. Therefore, sensitive detection of 4-chlorophenol is of great significance. Figure 6A shows the ECL signals of Ru (bpy)_3_^2+^/TPrA in the presence of different concentrations of 4-chlorophenol. A linear dependence between the reduced ECL intensity (Δ*I*_ECL_) and the concentration of 4-chlorophenol (*c*_4-CP_) is obtained with a range from 0.1 to 20 μM (Δ*I*_ECL_ = 734.9*c*_4-CP_ + 43.2, *R* ^2^ = 0.9982, Figure 6B). The limit of detection (LOD) is calculated to be 30 nM (*S*/*N* = 3). In addition, the ECL signal is quite stable. The relative standard deviation of ECL intensity for three consecutive detections is less than 2.5%. Owing to the high active area of 3DG electrode, the sensitivity on VMSF/APTES/3DG is four times higher than that of the corresponding system on the planar electrode (VMSF/APTES/GCE). The selectivity of the sensor was evaluated by comparing the ECL intensity in the presence of 5 orders of magnitude higher concentrations of ions (Na^+^, K^+^, Cl^−^, NO_3_^−^) or bioactive molecules (e.g., glucose, urea). The ECL intensity remained more than 98%, indicating no remarkable interference and high selectivity.

### Electroluminescence Determination of Chlorpheniramine Using Signal-on Mode

**FIGURE 7:**
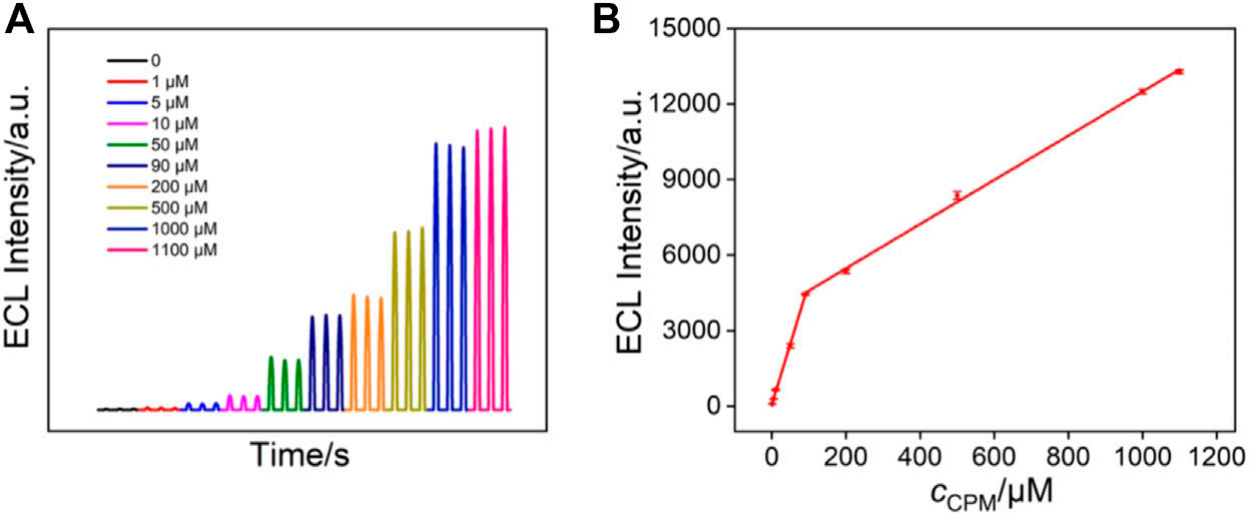
**(A)** ECL intensity of 10 μM Ru (bpy)_3_^2+^ in the presence of different concentrations of chlorpheniramine. **(B)** The linear dependence between the ECL intensity and the concentration of chlorpheniramine.

The ECL response of Ru (bpy)_3_^2+^ can be significantly enhanced in the presence of a co-reactant. Therefore, it is also possible to construct a signal-on ECL mode to detect co-reactants of Ru (bpy)_3_^2+^ (Liu and Zhou, 2006; Al-Hinaai et al., 2015). As a proof-of-concept demonstration, chlorpheniramine (CPM), which is one of the most effective antihistamines for the treatment of allergic and vasomotor rhinitis, allergic conjunctivitis, mild urticaria, angioedema, and anaphylactic shock, was detected by the VMSF/APTES/3DG sensor. Figure 7A shows the ECL signals of Ru (bpy)_3_^2+^ in the presence of different concentrations of chlorpheniramine. As seen, the ECL intensity of Ru (bpy)_3_^2+^ gradually increases with increasing the concentration of chlorpheniramine. A good linear correlation is found between the ECL intensity (*I*_ECL_) and the concentration of chlorpheniramine (*c*_CPM_) in the range of 1.0–90.0 μM (*I*_ECL_ = 48.15*c*_CPM _+ 78.54, *R* ^2^ = 0.9981, Figure 7B) and 90.0–1,100.0 μM (*I*_ECL_ = 8.78*c*_CPM _+ 3,714, *R* ^2^ = 0.9986). The LOD was calculated to be 430 nM (S/N = 3).

### Analysis of Real Samples

The application of VMSF/APTES/3DG in the detection of 4-chlorophenol in real environmental samples (lake water) was investigated (Supplementary Table S1 in Supplementary material). Since 4-chlorophenol was not detected in the lake water samples, the standard addition recovery method was used to evaluate the accuracy and reliability of the method. As seen, satisfactory recoveries (99.0–103%) and low RSD (<2.7%) for parallel detections were achieved, proving the high accuracy and reliability of detection in lake water. VMSF/APTES/3DG sensor was also applied to detect the content of chlorpheniramine in tablets. It is worth noting that the sensitivity in the tablet matrix is consistent with that obtained in the buffer (Supplementary Figure S5 in Supplementary material), indicating good anti-fouling characteristics of the sensor resulted from the VMSF nanochannels. The content of diphenhydramine in the tablets was determined to be 57.92 mg/g by the developed ECL sensor using linear extrapolation, which was quite similar with that (58.57 mg/g) obtained by standard high-performance liquid chromatography (HPLC) method.

## Conclusion

In summary, we have developed a novel 3D electrochemiluminescence platform based on monolithic and macroporous 3DG equipped with VMSF. In order to overcome the high hydrophobicity of 3DG and realize stable adhesion of VMSF, 3-aminopropyltriethoxysilane (APTES) is simply electrografted on 3DG as a hydrophilic molecular glue. The construction of the VMSF/APTES/3DG sensor is simple and convenient. Owing to the high conductivity and high active area of 3DG as well as the strong enrichment effect of VMSF on the ECL probe of Ru (bpy)_3_^2+^, VMSF/APTES/3DG exhibits significantly sensitized ECL signals. Using ECL signal-off and signal-on detection mode, 4-chlorophenol and chlorpheniramine can be sensitively detected as the proof-of-concept demonstrations. VMSF-modified 3DG might open a new way for the fabrication of 3D ECL sensors with high sensitivity and good anti-fouling performance.

## Data Availability

The original contributions presented in the study are included in the article/Supplementary Material. Further inquiries can be directed to the corresponding authors.

## References

[B1] Al-Hinaai M. Khudaish E. A. Al-Harthy S. Suliman F. O. (2015). A Solid-State Electrochemiluminescence Composite Modified Electrode Based on Ru(bpy)32+/PAHNSA: Characterization and Pharmaceutical Applications. Electrochim. Acta 176, 179–187. 10.1016/j.electacta.2015.06.148

[B2] Beluomini M. A. Stradiotto N. R. Boldrin M. V. (2021). Electrosynthesis of Three-Dimensional Nanoporous Nickel on Screen-Printed Electrode Used for the Determination of Narirutin in Citrus Wastewater. Food Chem. 353, 129427. 10.1016/j.foodchem.2021.129427 33714111

[B3] Chen K. Yao L. Su B. (2019). Bionic Thermoelectric Response with Nanochannels. J. Am. Chem. Soc. 141, 8608–8615. 10.1021/jacs.9b03569 31067855

[B4] Cheng S. Liu H. Zhang H. Chu G. Guo Y. Sun X. (2020). Ultrasensitive Electrochemiluminescence Aptasensor for Kanamycin Detection Based on Silver Nanoparticle-Catalyzed Chemiluminescent Reaction between Luminol and Hydrogen Peroxide. Sens. Actuators B: Chem. 304, 127367. 10.1016/j.snb.2019.127367

[B5] Ding H. Guo W. Su B. (2020). Imaging Cell‐Matrix Adhesions and Collective Migration of Living Cells by Electrochemiluminescence Microscopy. Angew. Chem. Int. Ed. 59, 449–456. 10.1002/anie.201911190 31631465

[B6] Ding H. Guo W. Zhou P. Su B. (2020). Nanocage-confined Electrochemiluminescence for the Detection of Dopamine Released from Living Cells. Chem. Commun. 56, 8249–8252. 10.1039/d0cc03370g 32558869

[B7] Dong L. Wei G. Cheng T. Tang J. Ye X. Hong M. (2020). Thermal Conductivity, Electrical Resistivity, and Microstructure of Cu/W Multilayered Nanofilms. ACS Appl. Mater. Inter. 12, 8886–8896. 10.1021/acsami.9b21182 31971777

[B8] Duan S. Peng J. Cheng H. Li W. Jia R. Liu J. (2021). A Label-free and Homogenous Electrochemical Assay for Matrix Metalloproteinase 2 Activity Monitoring in Complex Samples Based on Electrodes Modified with Orderly Distributed Mesoporous Silica Films. Talanta 231, 122418. 10.1016/j.talanta.2021.122418 33965055

[B9] Gamero-Quijano A. Dossot M. Walcarius A. Scanlon M. D. Herzog G. (2021). Electrogeneration of a Free-Standing Cytochrome C-Silica Matrix at a Soft Electrified Interface. Langmuir 37, 4033–4041. 10.1021/acs.langmuir.1c00409 33761740 PMC8562870

[B10] Garbayo I. Santiago A. Judez X. de Buruaga A. S. Castillo J. Muñoz-Márquez M. A. (2021). Alumina Nanofilms as Active Barriers for Polysulfides in High-Performance All-Solid-State Lithium-Sulfur Batteries. ACS Appl. Energ. Mater. 4, 2463–2470. 10.1021/acsaem.0c03032

[B11] Gu W. Wang H. Jiao L. Wu Y. Chen Y. Hu L. (2020). Single‐Atom Iron Boosts Electrochemiluminescence. Angew. Chem. 132, 3562–3566. 10.1002/ange.201914643 31873976

[B12] Guo W. Lin X. Yan F. Su B. (2016). Vertically Ordered Silica Mesochannel Modified Bipolar Electrode for Electrochemiluminescence Imaging Analysis. ChemElectroChem 3, 480–486. 10.1002/celc.201500329

[B13] He M. Cao L. Li W. Chang X. Ren Z. (2021). α-MnO2 Nanotube@δ-MnO2 Nanoflake Hierarchical Structure on Three-Dimensional Graphene Foam as a Lightweight and Free-Standing Supercapacitor Electrode. J. Alloys Comp. 865, 158934. 10.1016/j.jallcom.2021.158934

[B14] Huang X. Xie L. Lin X. Su B. (2016). Permselective Ion Transport across the Nanoscopic Liquid/liquid Interface Array. Anal. Chem. 88, 6563–6569. 10.1021/acs.analchem.6b01383 27240714

[B15] Huang H. Xia L. Zhao Y. Zhang H. Cong T. Wang J. (2020). Three-dimensional Porous Reduced Graphene oxide/PEDOT:PSS Aerogel: Facile Preparation and High Performance for Supercapacitor Electrodes. Electrochim. Acta 364, 137297. 10.1016/j.electacta.2020.137297

[B16] Jiokeng S. L. Z. Tonle I. K. Walcarius A. (2019). Amino-attapulgite/mesoporous Silica Composite Films Generated by Electro-Assisted Self-Assembly for the Voltammetric Determination of Diclofenac. Sens. Actuators B: Chem. 287, 296–305. 10.1016/j.snb.2019.02.038

[B17] Li L. Yu B. Zhang X. You T. (2015). A Novel Electrochemiluminescence Sensor Based on Ru(bpy)32+/N-Doped Carbon Nanodots System for the Detection of Bisphenol A. Anal. Chim. Acta 895, 104–111. 10.1016/j.aca.2015.08.055 26454465

[B18] Li L. Chen Y. Zhu J.-J. (2017). Recent Advances in Electrochemiluminescence Analysis. Anal. Chem. 89, 358–371. 10.1021/acs.analchem.6b04675 27959507

[B19] Li G. Huang B. Pan Z. Su X. Shao Z. An L. (2019). Advances in Three-Dimensional Graphene-Based Materials: Configurations, Preparation and Application in Secondary Metal (Li, Na, K, Mg, Al)-Ion Batteries. Energy Environ. Sci. 12, 2030–2053. 10.1039/c8ee03014f

[B20] Liu Y. Zhou W. (2006). Determination of Chlorpheniramine and its Binding with Human Serum Albumin by Capillary Electrophoresis with Tris(2,2'-bipyridyl)Ruthenium(II) Electrochemiluminescence Detection. Anal. Sci. 22, 999–1003. 10.2116/analsci.22.999 16837753

[B21] Liu Z. Qi W. Xu G. (2015). Recent Advances in Electrochemiluminescence. Chem. Soc. Rev. 44, 3117–3142. 10.1039/c5cs00086f 25803228

[B22] Liu J. He D. Liu Q. He X. Wang K. Yang X. (2016). Vertically Ordered Mesoporous Silica Film-Assisted Label-free and Universal Electrochemiluminescence Aptasensor Platform. Anal. Chem. 88, 11707–11713. 10.1021/acs.analchem.6b03317 27807970

[B23] Ma C. Cao Y. Gou X. Zhu J.-J. (2020). Recent Progress in Electrochemiluminescence Sensing and Imaging. Anal. Chem. 92, 431–454. 10.1021/acs.analchem.9b04947 31679341

[B24] Nasir T. Zhang L. Vilà N. Herzog G. Walcarius A. (2016). Electrografting of 3-aminopropyltriethoxysilane on a Glassy Carbon Electrode for the Improved Adhesion of Vertically Oriented Mesoporous Silica Thin Films. Langmuir 32, 4323–4332. 10.1021/acs.langmuir.6b00798 27065214

[B25] Nasir T. Herzog G. Hébrant M. Despas C. Liu L. Walcarius A. (2018). Mesoporous Silica Thin Films for Improved Electrochemical Detection of Paraquat. ACS Sens. 3, 484–493. 10.1021/acssensors.7b00920 29338195

[B26] Nasir T. Vodolazkaya N. A. Herzog G. Walcarius A. (2019). Critical Effect of Film Thickness on Preconcentration Electroanalysis with Oriented Mesoporous Silica Modified Electrodes. Electroanalysis 31, 202–207. 10.1002/elan.201800533

[B27] Nasir T. Gamero-Quijano A. Despas C. Dossot M. Herzog G. Walcarius A. (2020). Signal Amplification by Electro-Oligomerisation for Improved Isoproturon Detection. Talanta 220, 121347. 10.1016/j.talanta.2020.121347 32928388

[B28] Qi B.-P. Zhang X. Shang B.-B. Xiang D. Qu W. Zhang S. (2017). A Facile Method to Sensitively Monitor Chlorinated Phenols Based on Ru(bpy)32+ Electrochemiluminescent System Using Graphene Quantum Dots as Coreactants. Carbon 121, 72–78. 10.1016/j.carbon.2017.05.045

[B29] Qin Y. Wang Z. Xu J. Han F. Zhao X. Han D. (2020). Carbon Nitride Quantum Dots Enhancing the Anodic Electrochemiluminescence of Ruthenium(II) Tris(2,2′-Bipyridyl) via Inhibiting the Oxygen Evolution Reaction. Anal. Chem. 92, 15352–15360. 10.1021/acs.analchem.0c02568 33170643

[B30] Ullah W. Herzog G. Vilà N. Brites Helú M. Walcarius A. (2020). Electrochemically Assisted Deposition of Nanoporous Silica Membranes on Gold Electrodes: Effect of 3‐Mercaptopropyl(trimethoxysilane) "Molecular Glue" on Film Formation, Permeability and Metal Underpotential Deposition. ChemElectroChem 8, 142–150. 10.1002/celc.202001347

[B31] Ullah W. Herzog G. Vilà N. Walcarius A. (2021). Electrografting and Electropolymerization of Nanoarrays of Pani Filaments through Silica Mesochannels. Electrochemistry Commun. 122, 106896. 10.1016/j.elecom.2020.106896

[B32] Wang Z.-M. Peng W. Takenaka Y. Yoshizawa N. Kosuge K. Wang W. (2017). Sandwich-type Nanocomposite of Reduced Graphene Oxide and Periodic Mesoporous Silica with Vertically Aligned Mesochannels of Tunable Pore Depth and Size. Adv. Funct. Mater. 27, 1704066. 10.1002/adfm.201704066

[B33] Wang Y. Guo W. Yang Q. Su B. (2020). Electrochemiluminescence Self-Interference Spectroscopy with Vertical Nanoscale Resolution. J. Am. Chem. Soc. 142, 1222–1226. 10.1021/jacs.9b12833 31913616

[B34] Xi F. Zhao D. Wang X. Chen P. (2013). Non-enzymatic Detection of Hydrogen Peroxide Using a Functionalized Three-Dimensional Graphene Electrode. Electrochem. Commun. 26, 81–84. 10.1016/j.elecom.2012.10.017

[B35] Xi F. Xuan L. Lu L. Huang J. Yan F. Liu J. (2019). Improved Adhesion and Performance of Vertically-Aligned Mesoporous Silica-Nanochannel Film on Reduced Graphene Oxide for Direct Electrochemical Analysis of Human Serum. Sens. Actuators B: Chem. 288, 133–140. 10.1016/j.snb.2019.02.115

[B36] Xiao Y. Xu L. Li P. Tang X.-C. Qi L.-W. (2017). A Simple Microdroplet Chip Consisting of Silica Nanochannel-Assisted Electrode and Paper Cover for Highly Sensitive Electrochemiluminescent Detection of Drugs in Human Serum. Anal. Chim. Acta 983, 96–102. 10.1016/j.aca.2017.06.014 28811034

[B37] Yan F. Su B. (2016). Tailoring Molecular Permeability of Nanochannel-Micelle Membranes for Electrochemical Analysis of Antioxidants in Fruit Juices without Sample Treatment. Anal. Chem. 88, 11001–11006. 10.1021/acs.analchem.6b02823 27774789

[B38] Yan F. Lin X. Su B. (2016). Vertically Ordered Silica Mesochannel Films: Electrochemistry and Analytical Applications. Analyst 141, 3482–3495. 10.1039/c6an00146g 26952742

[B39] Zhang J. Jin R. Jiang D. Chen H.-Y. (2019). Electrochemiluminescence-based Capacitance Microscopy for Label-free Imaging of Antigens on the Cellular Plasma Membrane. J. Am. Chem. Soc. 141, 10294–10299. 10.1021/jacs.9b03007 31180678

[B40] Zhou Z. Guo W. Xu L. Yang Q. Su B. (2015). Two Orders-Of-Magnitude Enhancement in the Electrochemiluminescence of Ru(bpy)32+ by Vertically Ordered Silica Mesochannels. Anal. Chim. Acta 886, 48–55. 10.1016/j.aca.2015.06.005 26320635

[B41] Zhou L. Ding H. Yan F. Guo W. Su B. (2018). Electrochemical Detection of Alzheimer's Disease Related Substances in Biofluids by Silica Nanochannel Membrane Modified Glassy Carbon Electrodes. Analyst 143, 4756–4763. 10.1039/c8an01457d 30207331

[B42] Zhou P. Yao L. Chen K. Su B. (2020). Silica Nanochannel Membranes for Electrochemical Analysis and Molecular Sieving: a Comprehensive Review. Crit. Rev. Anal. Chem. 50, 424–444. 10.1080/10408347.2019.1642735 31352789

[B43] Zhou P. Yao L. Su B. (2020). Fabrication, Characterization, and Analytical Application of Silica Nanopore Array-Modified Platinum Electrode. ACS Appl. Mater. Inter. 12, 4143–4149. 10.1021/acsami.9b20165 31886640

[B44] Zu Y. Bard A. J. (2000). Electrogenerated Chemiluminescence. 66. The Role of Direct Coreactant Oxidation in the Ruthenium Tris(2,2')bipyridyl/Tripropylamine System and the Effect of Halide Ions on the Emission Intensity. Anal. Chem. 72, 3223–3232. 10.1021/ac000199y 10939391

