# Supplementary material for: Vertically Ordered Mesoporous Silica-Nanochannel Film-Equipped Three-Dimensional Macroporous Graphene as Sensitive Electrochemiluminescence Platform
Source: Front Chem. 2021 Nov 22;9:770512. doi: 10.3389/fchem.2021.770512 (PMC8645553; doi:10.3389/fchem.2021.770512)
Supplement: Supplementary file 1 [file Table1.DOCX]

SUPPORTING INFORMATION

**TABLES**

**Table S1.** Determination of 4-chlorophenol in lake water.

| Sample | Added  (μM) | Found  (μM) | RSD (%) | Recovery (%) |
| --- | --- | --- | --- | --- |
|  |  |  |  |  |
| Lake  water | 1.00 | 0.99 | 1.8 | 99.0 |
|  | 3.00 | 3.03 | 2.2 | 101 |
|  | 5.00 | 5.13 | 2.7 | 103 |
| **FIGURES**  **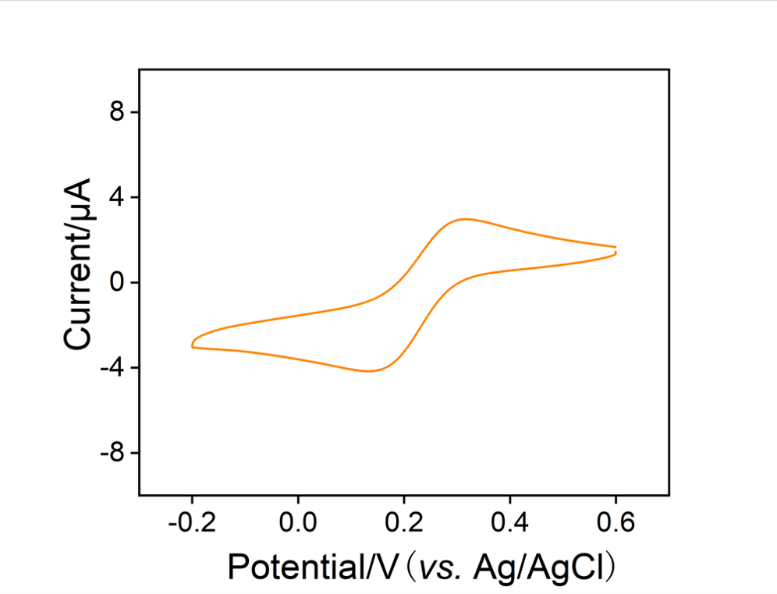**  **Fig. S1.** CV curve obtained at SM@VMSF/3DG in 0.05 M KHP solution containing 0.5 mM K_3_[Fe(CN)_6_].  ***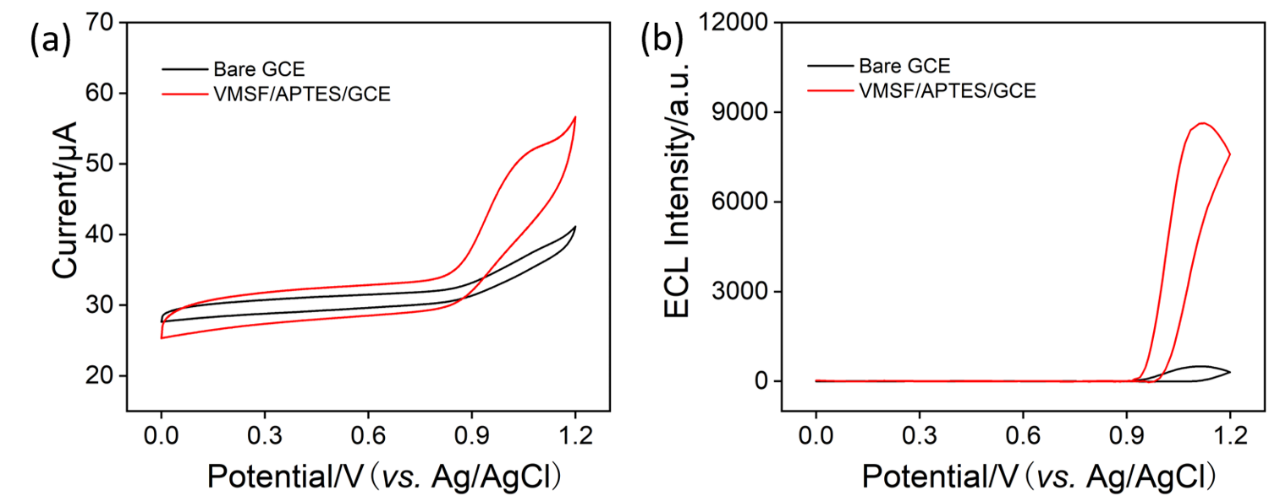***  **Fig. S2.** (a) CV and (b) ECL curves obtained at bare GCE and VMSF/APTES/GCE in PBS (0.01 M, pH 7.4) containing Ru(bpy)_3_^2+^ (10 μM) and TPrA (3 mM). The scan rate was 100 mV s^-1^.  **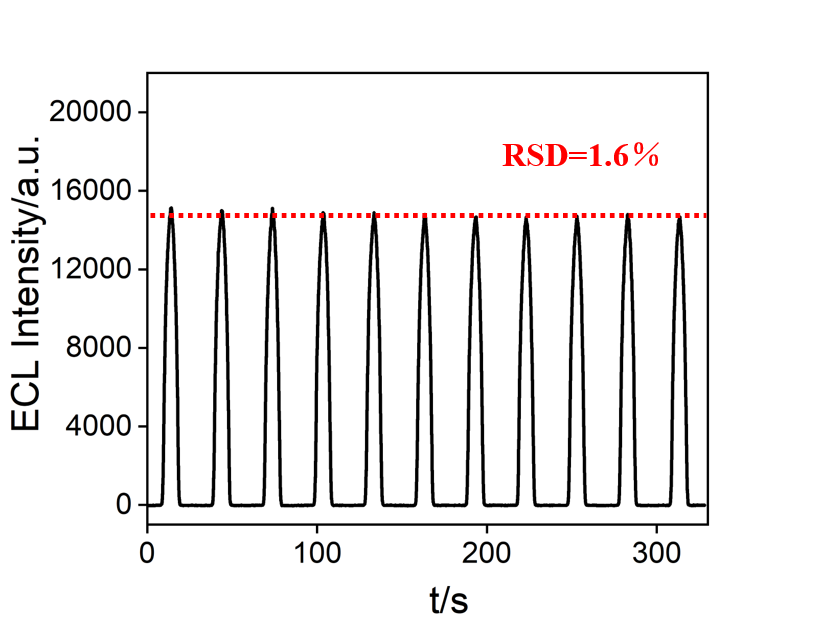**  **Fig. S3.** Time-dependent ECL signals of VMSF/APTES/3DG electrode in PBS (0.01 M, pH 7.4) containing Ru(bpy)_3_^2+^ (10 μM) and TPrA (3 mM) during successive CV scans.  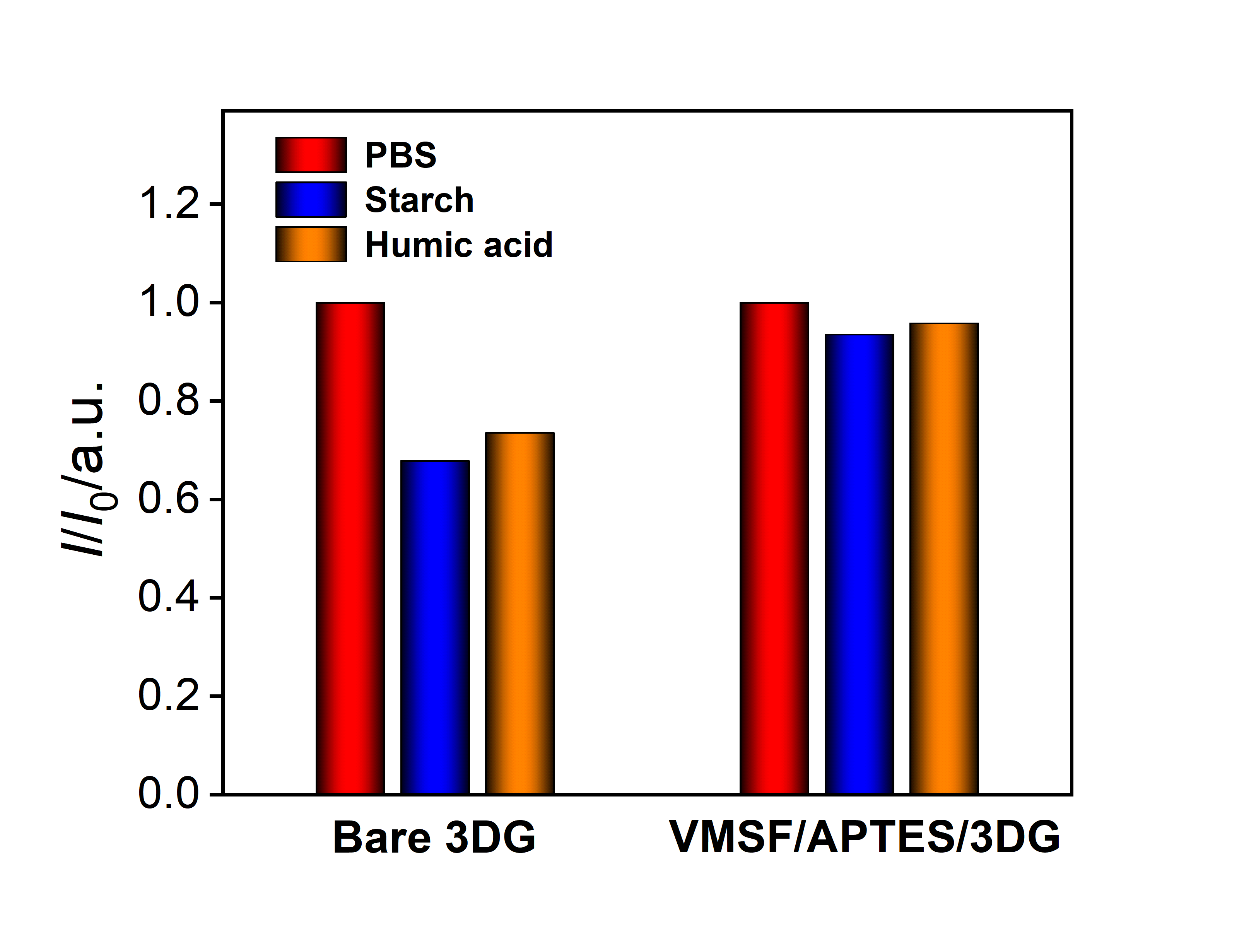  **Fig. S4.** The ratio of ECL intensity on bare 3DG or VMSF/APTES/3DG before (*I*_0_) and after (*I*) adding starch or humic acid. The concentration of starch or humic acid was 0.1 mg/ml. | | | | |


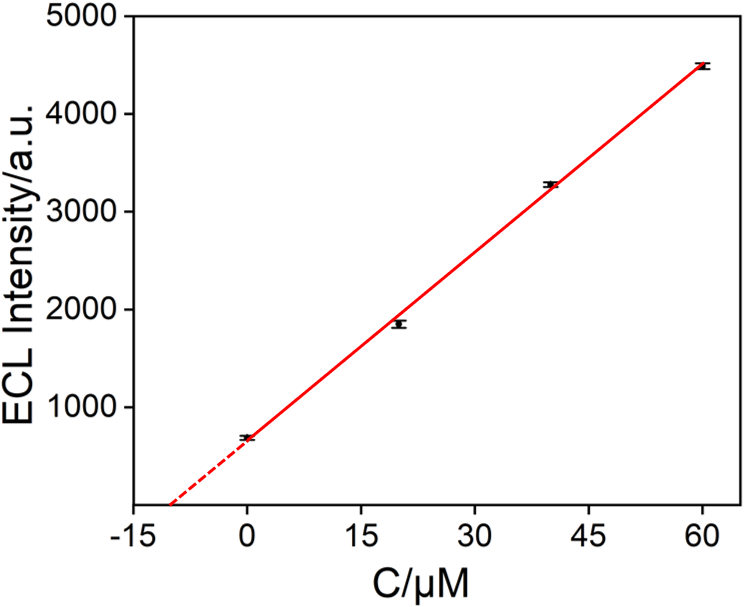


**Fig. S5.** The linear dependence between the ECL intensity and the concentration of chlorpheniramine in the analysis of chlorphenamine tablets.
